# Supplementary material for: Patterns of Intron Gain and Loss in Fungi
Source: PLoS Biol. 2004 Nov 30;2(12):e422. doi: 10.1371/journal.pbio.0020422 (PMC532390; doi:10.1371/journal.pbio.0020422)
Supplement: Table S1 — Also available at http://genes.mit.edu/NielsenEtAl/. (4.3 MB ZIP). [file pbio.0020422.st001.zip › NielsenEtAl/html/324.html]

AN1380.1.NCU07451.1.MG01046.1.FG08614.1


```
 CLUSTAL W (1.82) Multiple Sequence Alignments - Introns Inserted


Sequence 1: NCU07451.1	663 aa
Sequence 2: MG01046.1	700 aa
Sequence 3: FG08614.1	673 aa
Sequence 4: AN1380.1	658 aa
Alignment Length: 709 aa
Number Identitical Residues: 284 aa
Alignment Score (without introns) 15667


MG01046.1 	MAADSKPKLPQDGQENILVTSALPYVNNVPHLGNIIGSVL0SADVFAR~YCRAKGLQTLY
NCU07451.1	MASKNGPVLPQPGKENILITSALPYVNNVPHLGNIIGSVL0SADVFAR~FCRARGLPTLY
FG08614.1 	MASKS-PILPVEGKRNILVTSALPYVNNIPHLGNIIGSVL~SADVFSR~YCKARGYNTIY
AN1380.1  	MATEQ-KILPKKGERNILVTSALPYVNNVPHLGNIVGSVL~SADVFAR2YHKACGHPTLY
          	**:..   **  *:.***:*********:******:**** *****:* : :* *  *:*

MG01046.1 	VCGSDEYGTATETKALEEGVDPATLCAKYHAVHKEIYDWFRIEFDVFGRTPTPEHTQIVQ
NCU07451.1	ICGSDEYGTATETKALSEGVDPATLCAKYHALHKDVYDWFRISFDKFGRTPTDEHTGITQ
FG08614.1 	ICGSDEYGTATETKALEEGLSPADLCAKYHALHKGVYDWFRIDFDIFGRTPTQQQTQIVQ
AN1380.1  	ICGTDEYGTATETKALEEKVTPEELCAKYNKIHQEVYEWFEIGFDHFGRTPTQKHTEISQ
          	:**:************.* : *  *****: :*: :*:**.* ** ****** ::* * *

MG01046.1 	DIFSKLWKNGFIEQQETVQAFCARPEHNTFLADRFVEGECSLCHYTGARGDQ0CDACGNL
NCU07451.1	EIFAQLWKNGFIEERETTQAFCAVPEHNTFLADRYVEGECSICHDLGARGDQ~CDACGNL
FG08614.1 	QIFKELWKNGYIEERETTQPFCTHANHGKFLADRFVEGECSICHDLGARGDQ~CDACGGL
AN1380.1  	AIFKRLYDNGYLAEKTAEQPYCET--HGSFLADRYVEGECPRCHYDDARGDQ~CDKCGHL
          	 ** .*:.**:: :: : *.:*    *..*****:*****. **  .***** ** ** *

MG01046.1 	LDPMEPDKDSSAPAEDD--AQKATGWLINPKCKVDGSTPEKRRTRHLFLRLDALKDEIVS
NCU07451.1	LDPMQPDKN---AAGED--ESKGTGWLINPRCKLDGSTPERRKTKHLYIRLDELNKEITA
FG08614.1 	LDPFEPEREPS-ASEDDHVEAKATGFLINPRCKVDGTTPEKRKTKHLFLRLDAVKDLLVP
AN1380.1  	LDPFD---------------------LINPRCKLDGATPVKRDTKHIHLLLDKLQPEIEK
          	***::                     ****:**:**:** :* *:*:.: ** ::  :  

MG01046.1 	WLKET--ETGWSSNCVSITHSWIDKGLKPRGISRDLKWGVPI1PKGLDGLDEEEYARKVF
NCU07451.1	WLDRV--ENGWSANCVAITRSWINNGLKPRGITRDLKWGVPI1PKGLEGLSDEEYANKVF
FG08614.1 	WFHKSSKEGDWSANCISITQSWIDKGLLPRGITRDLKWGVPI~PQDLEGLSNEEYAEKVF
AN1380.1  	YVLPAMEKGEWPKNSRNITENWLREGLKDRGITRDLKWGVPV~P--LDGFDN-----KVL
          	:.    .:  *. *.  **..*: :**  ***:********: *  *:*:.:     **:

MG01046.1 	YVWFDACI1GYPSITKTYTDKDNLDGNNWEKWWKNPENVKLYQFMGKDNVP2FHTIIFPA
NCU07451.1	YVWFDACI~GYASITKCYTDEGNLEGRNWEKWWKNPDNVTLYQFMGKDNVP2FHTIIFPG
FG08614.1 	YVWFDACI~GYVSITKNLVDGDNLDGTNWEKWWKNPENVKLYQFMGKDNVP2FHTIIFPA
AN1380.1  	YVWFEACI~GYPSITANYTP-------DWELWWRNPEDVQLYQFLGKDNVP~FHSVIFPG
          	****:*** ** ***   .        :** **:**::* ****:****** **::***.

MG01046.1 	SQLGTRQPWTKVNHLSTTEYLNYEGGKFSKSKGVGVFGNTAQSTGIDPDVWRYYLLSRRP
NCU07451.1	TQLGTKEKWTQVNHLSTTEYLNYEGGKFSKSKGVGVFGNNARDTGIDPDVWRYYLLSRRP
FG08614.1 	SQLGTGENWTKVHKMSTTEYLNYEGGKFSKSRGVGVFGNTAKETGVDPDVWRYYLLSRRP
AN1380.1  	CQLGTKEKWTMLHHLNTAEYLNYEGGKFSKSRGIGVFGNNAKETGVAPDVWRYYLLKNRP
          	 **** : ** ::::.*:*************:*:*****.*:.**: *********..**

MG01046.1 	ESSDSEFKWE2EFIDANNNDLLKNLGNLVQRVVKFAHAKMGAKVPEYAKKEVPTFDQHKA
NCU07451.1	ESGDSEFKWE~EFIDSNNNDLLKNLGNLCQRVVKFCQAKLDAKVPEYPKTPIASLDEYKE
FG08614.1 	ETADSEFKWQ~EFIDCNNNDLLKNLGNLNQRILKFAQLKLDGVVPDYTKYTDERLEEHKK
AN1380.1  	ETGDTQFEWR~SFVDSNNSELLAKLGNLVNRVVKLVAASYGSTIPDFTVP--ESFQPTLD
          	*:.*::*:*. .*:*.**.:** :**** :*::*:   . .. :*::.      ::    

MG01046.1 	EVNKLLQDYISNLKATKLRAGLATTML2ISALGNKLLQDHKLGNQLLDEDPELCNGLIAL
NCU07451.1	EINKLLKEYNEQVKGTKLRAGLATVLS~ISAVGNKLLQDNKLSNQLLTEEPERCNAVINF
FG08614.1 	EVNEALKTFIAQFDAIKLRAGLASVMH~ISALGNKLLQDSKLNNQLLAEEPERCAAVIGL
AN1380.1  	EVTGLLRQYVEEMEGVHLRAGVSTAMK~IAEAGNGLIQANRLDNSLIANEPERAAAVVGT
          	*:.  *: :  :... :****:::.:  *:  ** *:*  :*.*.*: ::** . .::  

MG01046.1 	ALDHLYLIASLLFPYMPGTARSILRQIGLAKGDESQDSAFRIPEVWKGDSLTPGTPLGEP
NCU07451.1	ALNLLHLIAGVLSPYMPGTAQSILRQVGLKAAEGETDIPVVIPDTWTADALKAGEPIGTP
FG08614.1 	GINHLQLLASIVYPYMPSTSDAILEQIGSPG-------LVSIPETWTGDIVKPGQKIGEP
AN1380.1  	VLNLIHLLASVFAPYLPATSKSINEQLNAQFALIP--SLEDIKDGWKPVSLKPGHKIGKA
          	 :: : *:*.:. **:*.*: :* .*:.   .         * : *.   :..*  :* .

MG01046.1 	ERPFAQIPASKLEEWREAFGGEELRKQK-QIEAEKAAAKKLAREKDKEKKRLKKEAAKAA
NCU07451.1	ELLFSQIPAAKLEEWREAYGGEELRKQK-AIEAEKAAAKKLQREKEKEKKKAKKEKERLA
FG08614.1 	KLLFTQIPASKLDEWREAFGGEEIRKQK-ALEAEKAAAKKAAKDKKKQKKLALRQAEKAG
AN1380.1  	QYLFSRIDPKKADIWRDQFGGSQADRQKKEEEAAKAAAKKAAKDAAKAKKKEKKAKEAAA
          	:  *::* . * : **: :**.:  :**.  ** ******  ::  * **   :     .

MG01046.1 	TASGG---TTTGGGPG-STPTDLPLRPAANAGGAPAPGTPAAEGTSSQLEKAVEQKT
NCU07451.1	KEQAA---KEAAAG----ETKELPLR-------------------------------
FG08614.1 	EASTE---QKPAEEKKPVEATAVDLLPDDKPSET-----------------------
AN1380.1  	AAAATGAGSVESTAKGGVESTGVTTEGKNDEAVEKVTDGVAQVTFPSS---------
          	     .:.   .       .. :      . .    ..  :    .:.
```
